# Efficacy of Serology Testing in Predicting Reinfection in Patients With SARS-CoV-2

**DOI:** 10.1017/dmp.2020.216

**Published:** 2020-06-24

**Authors:** Rahul Chaturvedi, Ramana Naidu, Samir Sheth, Krishnan Chakravarthy

**Affiliations:** Division of Pain Medicine, Department of Anesthesiology, University of California San Diego, La Jolla, CA; California Orthopedics and Spine, Larkspur, CA; Sutter Roseville Pain Management, Roseville, CA; VA San Diego Health Care, San Diego, CA

**Keywords:** COVID-19, immunity, SARS-CoV-2, serology testing

## Abstract

In many parts of the United States, severe acute respiratory syndrome coronavirus 2 (SARS-CoV-2) cases have reached peak infection rates, prompting administrators to create protocols to resume elective cases. As elective procedures and surgeries get scheduled, ambulatory surgery centers (ASCs) must implement some form of widespread testing in order to ensure the safety of both the ASC staff and the patients being seen. The US Centers for Disease Control and Prevention (CDC) recently announced the approval of new serological testing for SARS-CoV-2, a test that can indicate the presence of IgM and IgG antibodies in the serum against viral particles. However, the possibility for reinfection raises questions about the utility of this new serological test, as the presence of IgG may not correspond to long-term immunity. SARS-CoV-2 has been known to form escape mutations, which may correspond to a reduction in immunoglobulin binding capacity. Patients who develop more robust immune responses with formation of memory CD8^+^ T-cells and helper CD4^+^ T-cells will be the most equipped if exposed to the virus, but, unfortunately, the serology test will not help us in distinguishing those individuals. Given the inherent disadvantages of serological testing, antibody testing alone should not be used when deciding patient care and should be combined with polymerase chain reaction testing.

Concerning reports released from the Korea Centers for Disease Control and Prevention (KCDC) have noted that up to 163 patients who were presumed to have recovered from severe acute respiratory syndrome coronavirus 2 (SARS-CoV-2) infection ended up testing positive with polymerase chain reaction (PCR) testing yet again.^1^ These patients tested positive after having tested negative on 2 different samples that were acquired within 24 hours of each other.^2^ Additional reports have also reported positive PCR results for SARS-CoV-2 following a presumed recovery.^3-5^ One possible explanation for testing positive after a previously negative result could be that the initial negative results that signified patient recovery were actually false-negative results, as false-negative rates have been reported to be as high as 30% for SARS-CoV-2 PCR testing.^6^ An alternative, albeit less plausible, reason includes the possibility of contamination of the samples, but most testing centers are requiring testers to change personal protective equipment (eg, gloves, gowns, masks) in between patients. One of the main points to consider is the basis of PCR testing – the test relies on amplifying nucleic acid in the sample, not fully active viral particles. There are numerous studies that have shown that the presence of inactive viral RNA outlasts infectious viral particles in the body.^7,8^ While the immune system generates antibody responses to the surface protein of viral particles, the genetic material (RNA, DNA) left behind degrades over time.^9^ Thus, positive PCR results after recovery may not necessarily signify reinfection, but rather the presence of leftover genetic material from previously active infection. Wolfel et al. isolated the live virus from individuals infected with SARS-CoV-2 but noticed that, after Day 8 of infection, the live virus was not able to be isolated, despite high overall viral loads.^10^ This concept is further strengthened by Zhang et al., who reported a case series on 6 patients who tested positive for SARS-CoV-2 through nasopharyngeal or rectal PCR testing after previously reported a recovery.^11^ Despite positive PCR test results, all patients in the study were asymptomatic and had unchanged clinical imaging, indicating that the presence of a positive PCR result does not necessarily signify reinfection and fails to correlate clinically.

However, the KCDC determined recovery as 2 separate negative PCR results within 24 hours. For patients to test positive after having 2 consecutive negative results, this would require 2 previous consecutive false-negative results or an increase in viral genetic material, possibly secondary to reinfection. The possibility for reinfection raises questions about the utility of the new serology tests approved by the US CDC. Does the presence of IgG truly infer long-term immunity, and, moreover, can health care providers truly use it to be confident in decision-making? There are 3 main mechanisms for reinfection; the immune response can be ineffective, strain-specific, or short-lived. Monoclonal antibodies formed against the SARS-CoV-2 virus target the Spike (S) glycoprotein component, the receptor-binding domain of the virus. SARS-CoV-2, however, has been shown to develop “escape mutants,” or alterations, in the epitope of the S protein that contribute to host tropism and viral virulence. Sui et al. noted that major variations exist in the S protein at positions 360, 479, and 487.^12^ The group found that by altering 1–2 amino acids at those positions, previously efficacious neutralizing antibodies to SARS-CoV-2 led to a 20–50% reduction in binding capacity. Theoretically, if SARS-CoV-2 is also able to form “escape mutants” in the S protein, IgG antibodies formed in patients may be less ineffective, though not completely, in neutralizing the virus. This could mean that patients continue to remain resistant to SARS-CoV-2 infection even after mutations, with antibody responses that are 50–80% efficacious.

As described previously, another component of whether a patient can be reinfected is dependent on the duration of the body’s immune response. Barthold et al. found that the mechanism by which 2 groups of mice were inoculated with a murine coronavirus species impacted the duration of conferred resistance, despite both groups having similar antibody responses.^13^ Immunoglobulins alone, therefore, are not truly sufficient to confer long-term immunity to coronavirus. The presence of CD4^+^ T-cells and memory CD8^+^ T-cells, which produce effector cytokines and IFN-γ, has been shown to be vital in providing protection from coronavirus.^14^ Previous studies have shown that virus-specific memory CD8^+^ T-cells were found to persist for up to 6 years after a SARS-associated coronavirus infection, but memory B-cells and accompanying antibodies were undetectable at that time.^15^

Serology testing thereby continues to remain in question. In the short term, patients who test positive for IgG certainly may have a level of resistance to SARS-CoV-2, albeit possibly with reduced efficacy if escape mutations were to arise. A year from now, the presence of IgG may not be sufficient to assume immunity in a patient; those with more robust immune responses with formation of memory CD8^+^ T-cells and helper CD4^+^ T-cells will be the most equipped if exposed to the virus; however, unfortunately, serology testing will not help us in distinguishing those individuals. Based on the previous findings, antibody testing alone should not be used when deciding patient care and should be combined with PCR testing.
